# Pediatric Sternal Segment Dislocation Caused by Different Types of Injury: A Report of Two Cases

**DOI:** 10.7759/cureus.94244

**Published:** 2025-10-09

**Authors:** Kosuke Shintani, Takayoshi Matsushita, Chinatsu Ohira, Yuma Onishi, Hidetomi Terai

**Affiliations:** 1 Orthopaedic Surgery, Osaka Metropolitan University Graduate School of Medicine, Osaka, JPN; 2 Pediatric Orthopaedics, Osaka City General Hospital, Osaka, JPN

**Keywords:** child trauma, conservative treatment, remodeling, sternal segment dislocations, sternal trauma, young children

## Abstract

Sternal segment dislocations in children are extremely rare, and there is currently no consensus on their mechanism of injury or management approach.

We report two pediatric cases of sternal segment dislocation with different mechanisms of injury. Case 1, a four-year-old girl, sustained an injury without any direct impact or bruising. Radiographic examination revealed dislocation of the first sternal segment with anterior displacement of the sternal body. Case 2, a seven-year-old boy, developed chest pain after striking his chest against the edge of a desk. Imaging showed no anterior displacement of the sternal body relative to the manubrium. Both cases were treated conservatively, resulting in spontaneous bone remodeling and favorable outcomes, despite surgical management being more commonly reported in the literature. Careful consideration is warranted when determining treatment strategies for young patients with significant potential for bone remodeling.

## Introduction

The sternum is stabilized by the sternocostal articulations and surrounding ligaments, making it relatively resistant to injury. In children, the sternum is even more flexible due to its multiple ossification centers, which gradually fuse from childhood to adulthood [1,2]. Consequently, sternal fractures and dislocations in adults are rare, and such injuries are even more unusual in children because of the increased flexibility of the pediatric sternum.

Among these injuries, sternal segment dislocation is extremely uncommon, and reports in the pediatric literature have remained limited since the early 1980s [3-5]. Reported clinical features typically include localized anterior chest pain and tenderness, while respiratory compromise is rare [5]. Diagnosis is most often achieved using plain radiographs, although cross-sectional imaging and ultrasonography have also been utilized in some cases [6].

Due to the limited number of published cases, the epidemiology, mechanisms, and optimal management of this condition remain uncertain. Although sternal segment dislocation has been managed both conservatively and surgically, the indications for each approach remain controversial. Furthermore, the mechanism of injury, whether caused by direct or indirect force, remains unclear, and several possibilities have been proposed. We report two cases of pediatric sternal segment dislocation caused by different mechanisms, both of which were successfully treated with conservative management.

## Case presentation

Case 1

**Figure 1:**
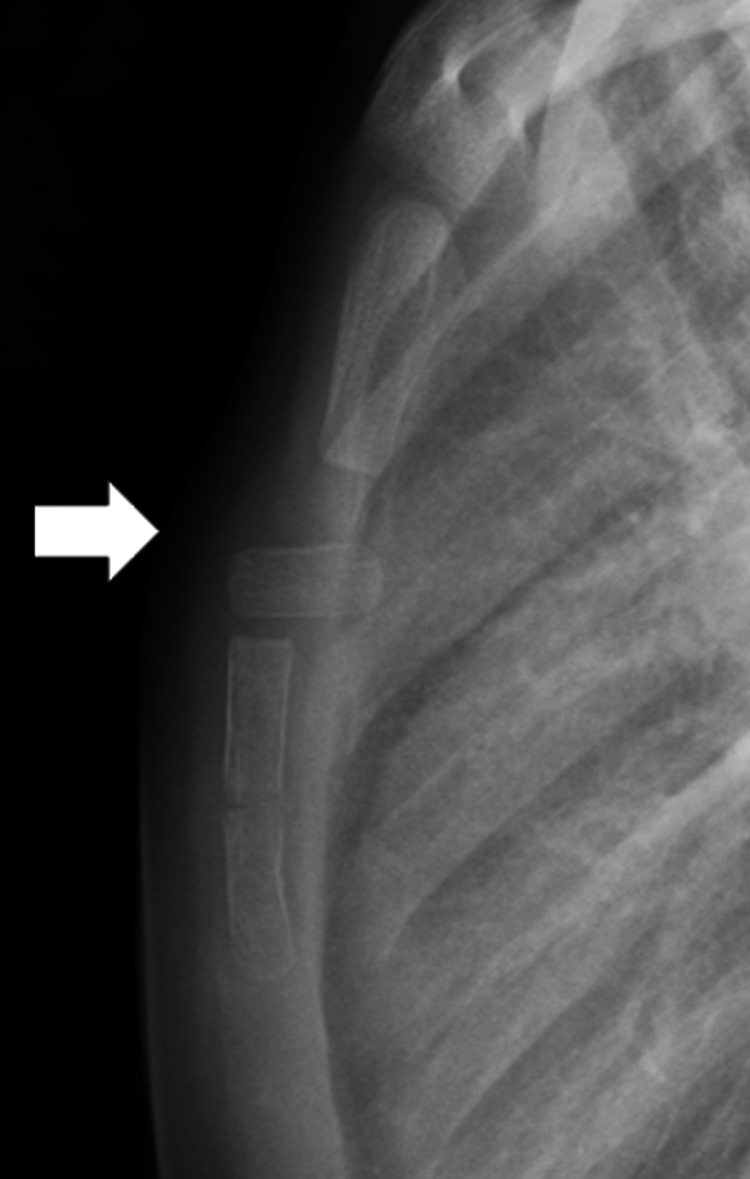
Anterior dislocation of the first sternal segment on radiograph (white arrow).

**Figure 2:**
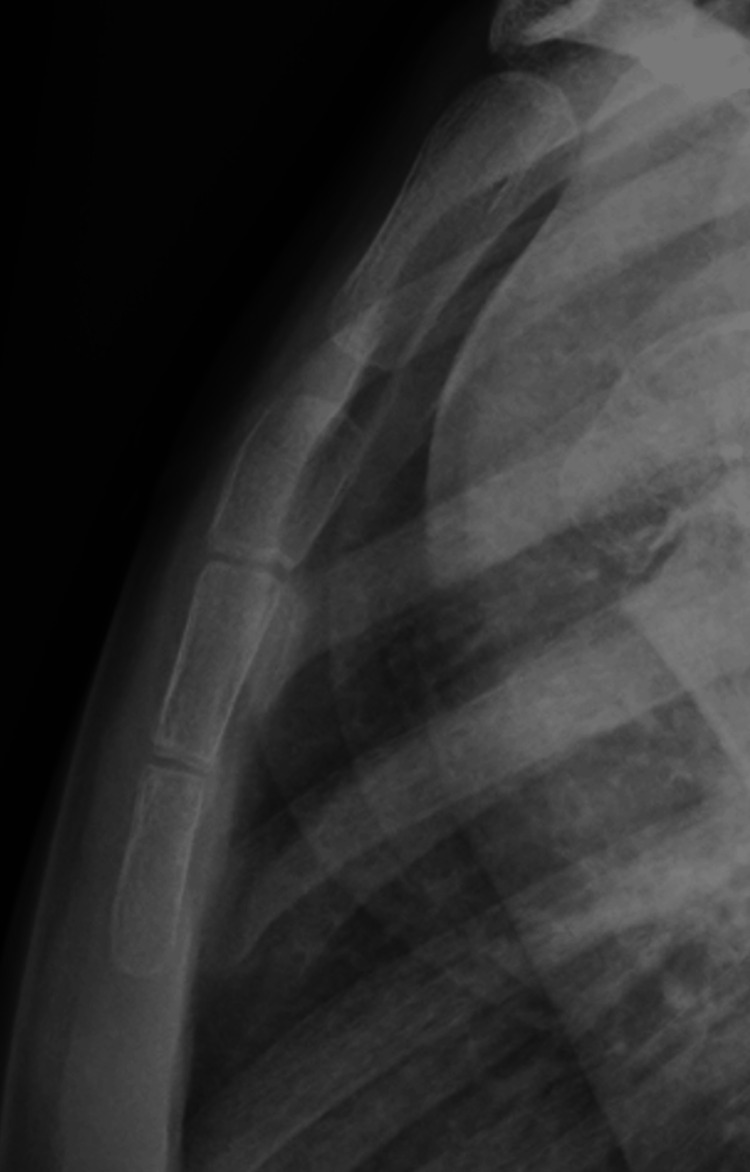
Four-year follow-up showing complete remodeling and resolution of the dislocation.

A four-year-old girl experienced chest pain after running and jumping outdoors, despite no history of impact or bruising. Ten days after the injury, she visited a nearby hospital because the pain persisted. An X-ray revealed a dislocated sternal segment, and she was referred to our hospital for further management. On physical examination, a slight bump, tenderness, and swelling were noted in the mid-sternal region. There were no bruises, abrasions, abnormal heart sounds, or dyspnea. Radiography showed anterior dislocation of the first sternal segment (Figure 1). We explained both conservative and surgical management options to the patient’s parents, who strongly preferred conservative treatment. No chest brace was applied; instead, physical activity was restricted for two months, and the patient was monitored at home without physiotherapy. The pain resolved within one month of the injury. Follow-up chest X-rays demonstrated new bone formation around the superior and inferior margins of the affected segment within three months. At the final follow-up, four years after the injury, radiographic remodeling was nearly complete, and the anterior dislocation had been reduced. No recurrence of pain was noted throughout the observation period (Figure 2).

Case 2

**Figure 3:**
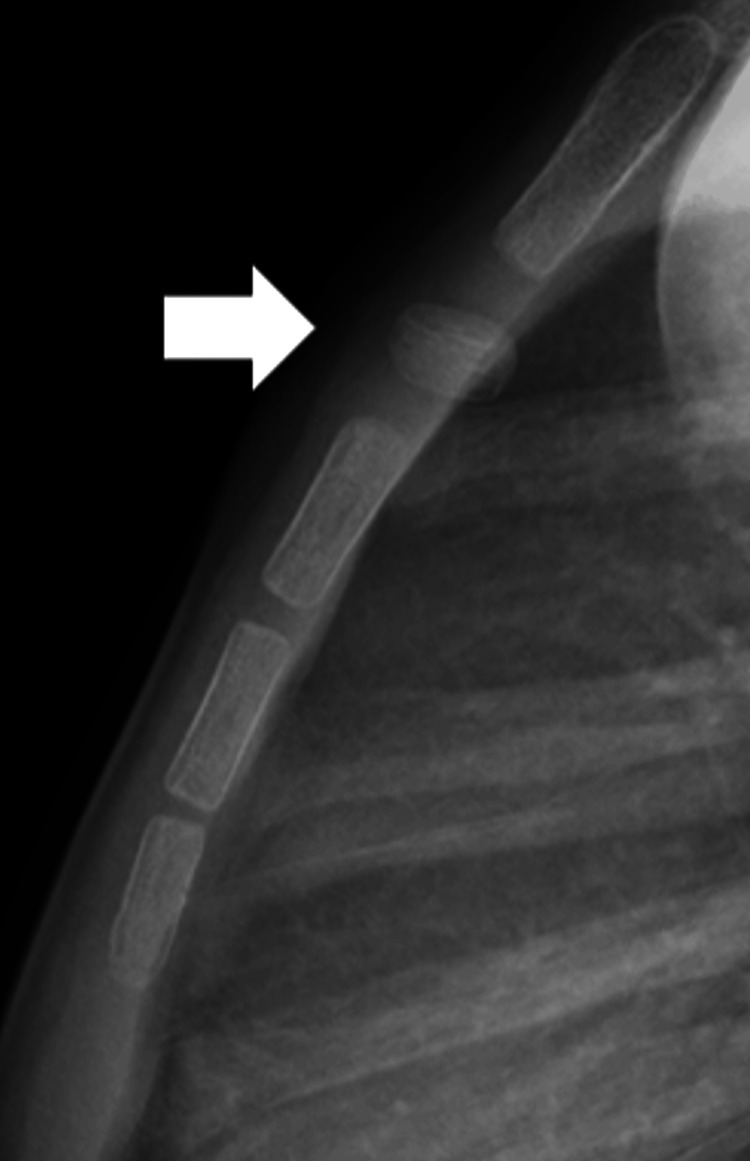
Sternal segment dislocation without anterior displacement on radiograph (white arrow).

**Figure 4:**
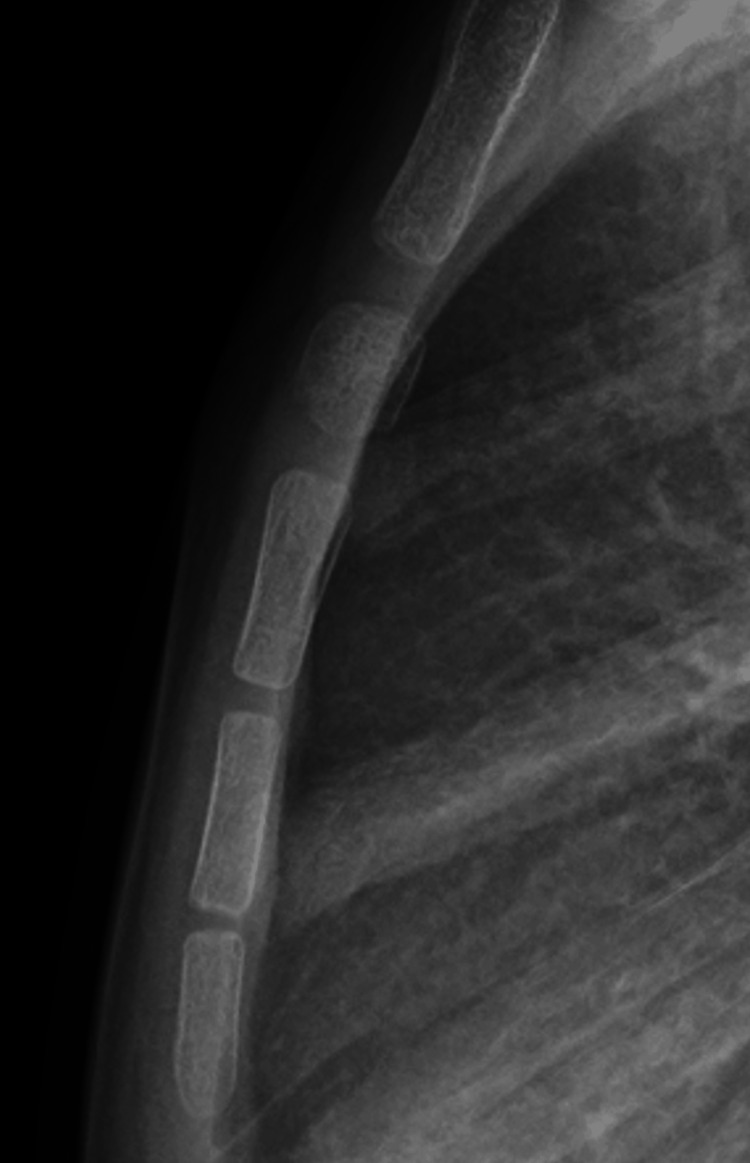
Two-year follow-up demonstrating successful remodeling of the sternal segment.

A seven-year-old boy experienced chest pain after striking his chest on the edge of a desk. He was initially kept under observation at home without medical consultation, despite the immediate onset of chest pain. Three weeks later, he visited a nearby clinic because the pain persisted. An X-ray revealed a dislocated sternal segment, and he was referred to our hospital one month after the injury. Radiography showed a dislocation of the first sternal segment of the sternal body, although no anterior displacement was present, unlike in Case 1 (Figure 3). By the time of presentation, the chest pain had already resolved. There were no abnormalities in heart sounds, breath sounds, or signs of dyspnea. Given that some time had passed since the injury and the symptoms had improved, conservative management was initiated, as in Case 1. The patient resumed normal activities one month after the injury and was cleared to participate in sports after three months. Follow-up X-rays demonstrated progressive new bone formation at the superior and inferior borders of the affected segment. At the final follow-up, two years post-injury, remodeling was nearly complete, and no recurrence of pain was observed (Figure 4).

## Discussion

We present two cases of pediatric sternal segment dislocation that were treated conservatively with good outcomes, each with a different mechanism of injury. These cases suggest that treating segmental sternal dislocation without surgical intervention is possible in patients with the capacity for bone remodeling, such as young children.

The pediatric sternum is anatomically composed of the manubrium and four ossification centers within the sternal body. The sternal segments begin to fuse in a craniocaudal direction at around seven years of age, a process that is typically complete by 25 years of age [1,2]. Nonunion and partial defects may occur in adulthood but are often without pathological significance [7,8]. Segmental sternal dislocation in children was first described in 1982 [3]. It is extremely rare because the pediatric sternum is more flexible and less prone to fracture than the adult sternum. Although there have been many reports since then, comprehensive reports are limited.

Previous reports have shown that symptoms mainly consist of anterior chest pain and tenderness at rest, with a few cases of respiratory distress. The age at onset has ranged from 18 months to 14 years [5]. Diagnosis is primarily based on plain X-rays, though there have been reports of diagnosis using CT scans and ultrasounds. We made the diagnosis using plain X-rays, and it does not appear to be difficult.

Two mechanisms of sternal segment dislocation have been postulated. The first type occurs when an external force is applied directly to the anterior chest. The second type occurs when severe flexion of the neck causes posterior-inferior traction via the second rib. This results in the sternum dislocating anteriorly relative to the manubrium. Anterior dislocation of the sternal body relative to the manubrium does not occur in cases of injury caused by direct external force, whereas it does occur in cases of injury caused by indirect external force [9,10].

Past reports indicate that, when both the mechanism of injury and imaging findings were confirmed, dislocation was not observed in some cases caused by direct external force [5,11-13], whereas the sternal body was displaced anteriorly relative to the manubrium in all cases caused by indirect force [6,14,15]. It was inferred that, depending on the magnitude and direction of the external force, anterior dislocation may or may not be detected. In fact, in Case 1, where there was no obvious mechanism of trauma, the sternal body was anteriorly dislocated. In contrast, in Case 2, where there was a mechanism of trauma, the sternal body was not anteriorly dislocated relative to the manubrium.

The treatment of segmental sternal dislocation includes conservative and surgical management. Since the first four cases were reported in 1982, subsequent reports have described surgical treatment [3,12]. Internal fixation materials, including metal plates and bioabsorbable pins, were used for fixation. Since then, several reports have documented positive outcomes with conservative treatment. However, there are also cases in which surgery has been performed. In most surgical treatments, good outcomes have been reported, although they are invasive and usually require plate removal in pediatric cases [5, 11, 16]. Similarly, favorable results have been reported in most conservatively managed cases, though specific methods and treatment recommendations have not been detailed [12,14,15]. Currently, there is no clear consensus on the most effective treatment method. In our two cases, the first ossification center rotated approximately 90°, which makes most surgeons eager to opt for surgical reduction and fixation. Nevertheless, good outcomes were achieved with conservative treatment in both cases, as evidenced by X-rays showing self-correction. Nijs and Broos reported a case in which pain did not improve with conservative treatment, resulting in surgery [16]. However, the conservative treatment lasted only two weeks after the injury occurred, and the condition may have improved with continued rest and observation. Even in previously reported cases that were treated surgically, one could argue that conservative care could have been an effective treatment option. Therefore, careful consideration is required when deciding on surgical treatment.

## Conclusions

We presented two cases of pediatric sternal segment dislocation that occurred via different mechanisms and were both successfully treated conservatively. In our cases, the pain resolved completely, and both patients were able to resume unrestricted activities. Long-term follow-up revealed progressive remodeling without recurrence or functional limitations.

When treating young children, it is important to carefully consider the potential for natural remodeling before opting for surgery. In many pediatric patients, careful observation and individualized management can be sufficient, and early recognition is essential for favorable outcomes.
